# Literary Notes

**Published:** 1898-03

**Authors:** 


### Literary Notes.

The Lofoten Islands, and their principal product, is the title of a handsomely-printed and illustrated brochure issued by Messrs. Parke, Davis & Co., of Detroit. It gives the details of the capture of codfish and the manufacture of cod-liver oil, and is full of interesting information on the subject. It will be furnished by the publishers on publication.

The *Monthly Cyclopedia of Practical Medicine* is the title of a magazine that is issued to replace the *Universal Medical Journal.* The new journal is a handsomely printed, double-columned, large octavo, and is edited by Dr. Charles E. Sajous, 2043 Walnut street, Philadelphia. In the introduction adequate reasons for the change are given and it is also announced that there will be important modifications made in the publication of the *Annual of the Universal Medical Sciences*, the first volume of which is to issue soon.

---

The Clyde Line of New York and Florida steamships has issued a handsome little brochure, beautifully illustrated, setting forth the pleasures and healthfulness of a winter trip to the south. To examine it makes one long for the land of the palms, roses and oranges. Physicians should not only send their patients but go themselves. It is one of the most healthful, restful, delightful and economical vacations that a physician can take. Information cheerfully furnished by the Journal.

